# The mediation effect of serum metabolites on the relationship between long-term smoking exposure and esophageal squamous cell carcinoma

**DOI:** 10.1186/s12885-021-08151-6

**Published:** 2021-04-15

**Authors:** Mengke Wei, Lihong Zhao, Jiali Lv, Xia Li, Guangshuai Zhou, Bingbing Fan, Xiaotao Shen, Deli Zhao, Fuzhong Xue, Jialin Wang, Tao Zhang

**Affiliations:** 1 Department of Biostatistics, School of Public Health, Cheeloo College of Medicine, Shandong University, PO Box 100, 44 Wenhua Xi Rd, Jinan, 250012 Shandong China; 2 Tumor Preventative and Therapeutic Base of Shandong Province, Feicheng People’s Hospital, Feicheng, 271600 China; 3 Shandong Cancer Hospital and Institute, Shandong First Medical University and Shandong Academy of Medical Sciences, Jinan, 250117 China; 4 Interdisciplinary Research Center on Biology and Chemistry, and Shanghai Institute of Organic Chemistry, Chinese Academy of Sciences, Shanghai, 200032 China

**Keywords:** Esophageal squamous cell carcinoma, Cigarette smoking, Metabolites, Mediation analysis

## Abstract

**Background:**

Long-term smoking exposure will increase the risk of esophageal squamous cell carcinoma (ESCC), whereas the mechanism is still unclear. We conducted a cross-sectional study to explore whether serum metabolites mediate the occurrence of ESCC caused by cigarette smoking.

**Methods:**

Serum metabolic profiles and lifestyle information of 464 participants were analyzed. Multiple logistic regression was used to estimate adjusted odds ratios (ORs) and 95% confidence intervals (CIs) of smoking exposure to ESCC risk. High-dimensional mediation analysis and univariate mediation analysis were performed to screen potential intermediate metabolites of smoking exposure for ESCC.

**Results:**

Ever smoking was associated with a 3.11-fold increase of ESCC risk (OR = 3.11, 95% CI 1.63–6.05), and for each cigarette-years increase in smoking index, ESCC risk increased by 56% (OR = 1.56, 95% CI 1.18–2.13). A total of 5 metabolites were screened as mediators by high-dimensional mediation analysis. In addition, glutamine, histidine, and cholic acid were further proved existing mediation effects according to univariate mediation analysis. And the proportions of mediation of histidine and glutamine were 40.47 and 30.00%, respectively. The mediation effect of cholic acid was 8.98% according to the analysis of smoking index.

**Conclusions:**

Our findings suggest that cigarette smoking contributed to incident ESCC, which may be mediated by glutamine, histidine and cholic acid.

**Supplementary Information:**

The online version contains supplementary material available at 10.1186/s12885-021-08151-6.

## Background

Esophageal squamous cell carcinoma (ESCC) remains the most predominant type of esophageal cancer, and is an important health problem in high-risk areas [1]. Epidemiological studies have found that risk factors for ESCC, including smoking, alcohol drinking, high-temperature foods, diet, and polycyclic aromatic hydrocarbons from a variety of sources [2]. It has been found that ESCC is more common in men (69%) than women (31%) [3] and the smoking exposure is more popular among men. Most studies have identified smoking as an important risk factor for ESCC in spite of the magnitude of the effect of smoking on ESCC varies [4, 5].

Metabolic alterations have been recognized as a key hallmark of cancer and metabolism-focused research has received renewed attention recently [6, 7]. So far, a number of case-control studies have found significantly changes in metabolic profile in ESCC patients [8–10], indicating that metabolites have the potential as biomarkers of ESCC. It is generally believed that metabolites will be influenced by environmental factors [11, 12], and there are many reports on cigarette smoking [13, 14]. Exposure biomarkers of smoking like nicotine and polycyclic aromatic hydrocarbons, were significantly higher in smokers than non-cigarette users [15, 16]. Cigarette smoking can also cause changes in other metabolites, for example, metabolites associated with inflammation and oxidative damage [17].

These evidences suggest that metabolites may be mediators in the development of ESCC caused by smoking. However, there is still a lack of relevant research at present. And previous studies were limited to smoking status at one point in time, but we believed that the effects of smoking are long-term, so we tend to pay more attention to the cumulative exposure of smoking. Our screening platform of ESCC has collected detailed information on smoking exposure and found many precancerous lesions, which provides the research basis and is of great significance for the prevention of ESCC [18, 19].

We hypothesized that serum metabolites were intermediate variables in the causal pathway between cigarette smoking and ESCC. And we aimed to assess the causal mechanism of metabolites in cigarette smoking and ESCC by high-dimensional mediation analysis and univariate mediation analysis.

## Methods

### Study population

Participants were collected at the Esophageal Cancer Screening Base of Shandong Province (City of Feicheng, Shandong, China) between July 2013 and April 2014. In this base, 779 participants aged 40–69 years were screened for esophageal cancer using endoscopy with mucosal iodine staining [20] and tissue biopsies were performed in the iodine-negative participants. The pathologic evaluation was performed by two pathologists according to the pathologic diagnostic criteria of early diagnosis and early treatment of upper gastrointestinal cancer. Participants involved in this study did not take any medications, surgery, radiotherapy or chemotherapy, and those suffering from metabolic diseases, liver diseases, kidney diseases or any other cancers were excluded. We excluded participants with inflammation (*n* = 80) and gastric lesions (*n* = 79) by the pathological diagnosis, and without smoking index-related information (*n* = 156). After these exclusions, data were available from 464 participants for the present analysis. Except for normal squamous epithelium (*n* = 257), most of them were precancerous lesions (mild atypical hyperplasia, 148; moderate atypical hyperplasia, 30; severe atypical hyperplasia, 11) and ESCC (tumor in situ, 7; squamous cell carcinomas with infiltrating, 10; intramucosal squamous cell carcinomas, 1). The precancerous stage was an important period for early prevention and control of ESCC development [21]. Thus, we defined normal squamous epithelium as the control group (*n* = 257), and precancerous lesions and cancer as the case group (*n* = 207).

The research was subject to approvals from the Ethics Committee of the Shandong Cancer Hospital and Institute, and all participants provided informed written consent.

### Measurements

Information on behavioral lifestyles was obtained in a questionnaire survey before pathological screening with mucosal iodine staining. Current smokers were defined as those who have smoked at least 100 cigarettes during their lifetime and who currently smoked every day or some days. Former smokers were defined as those who have smoked at least 100 cigarettes during their lifetime and who currently did not smoke at all. Non-smokers were those who never smoked in their life time. To indicate the long-term smoking status, former and current smokers were combined into ever smokers. Information about the number of cigarettes smoked per day and years of smoking was asked further to derive variable on the long-term smoking exposure. Cumulative smoking exposure was evaluated as smoking index, calculated by multiplying the number of cigarettes smoked per day by the number of years of smoking, and smoking index for non-smokers is equal to 0. Alcohol drinking status (yes and no), was self-reported at enrollment. Anthropometric measurements, including height and weight, were obtained by well-trained examiners, with the participants wearing light, thin clothing and no shoes. Body mass index (BMI) was calculated as the measured weight in kilograms divided by the square of the height in meters.

### Serum sample collection

All of the participants were fasting overnight, and 5 mL of peripheral venous blood was collected in the morning. The blood was then allowed to clot in a water batch of 37 °C for 30 min, and followed by centrifugation at 3000 rpm for 15 min. Then the serum supernatant was taken, immediately freezed in liquid nitrogen, and stored at − 80 °C until further analyses [18].

### UHPLC-QTOF/MS analysis

The serum samples were randomly injected for the ultra-high-performance liquid chromatography quadruple time-of-flight mass spectrometry analysis. Quality control (QC) samples were prepared by pooling aliquots of all serum samples that were representative of the serum samples under analysis and used for data normalization. ProteoWizard Software was used to convert mass spectrometry raw data (.d) files into mzXML format files. Through a series of pre-treatment, peak annotation, and normalization of the original data, a total of 8182 peaks were detected (see details in Supplement materials). The detailed information of the experiment has been described in our previous study [18]. 341 metabolites identified by in-house tandem MS spectral library or online databases and with relative standard error < 30% in QC samples were retained for subsequent analysis.

### Statistical analysis

Student’s t-tests (continuous variables) or *χ*^2^ tests (categorical variables) were performed to describe the baseline characteristics of 464 participants in each group of case and control. Multiple logistic regression was applied to evaluate the association between ever smoking (or smoking index) and ESCC risk by odds ratios (ORs) and 95% confidence intervals (CIs), by adjusting for age and gender. General linear regression model was used to assess the associations of smoking index with metabolites and metabolites with ESCC outcome. Models were adjusted for age and gender, with the false discovery rate (FDR) adjusted to account for multiple comparisons.

**Fig. 1:**
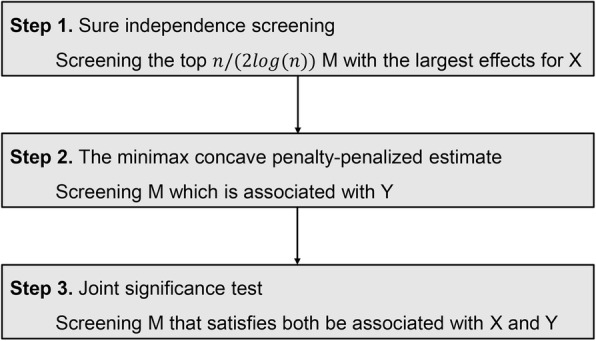
Flow diagram illustrating the analysis steps of high dimensional mediation analyses, where X refers to smoking exposure (ever smoking or smoking index), M refers to metabolite and Y refers to the ESCC outcome

High dimensional mediation analyses were then performed by R package *HIMA* to discover potential metabolic mediators for the association between ever smoking and ESCC risk [22, 23]. The analysis steps of this method are as follows (Fig. 1): 1) The sure independence screening was used to identify a subset of metabolites that are among the top *n*/(2 *log* (*n*)) largest effects of ever smoking on mediators whether *P* value makes sense or not, where n is the sample size [24]; 2) The minimax concave penalty was performed to evaluate the effects of metabolites subset on the ESCC outcome [25]; 3) The relevant FDR of ever smoking-metabolite and metabolite-ESCC associations based on a joint significance test were used to ensure intermediate metabolites. And potential mediation effects of metabolites on the association between smoking index and ESCC risk were explored by the same method.

To better understand the significance of these metabolites selected by high dimensional mediation analyses, we also performed univariate mediation analysis on selected intermediate metabolites, adjusted for age and gender, using the *medflex* package in R [26]. All statistical analyses were carried out using software R-project (V.3.6.0). We interpreted two-sided *P* values of < 0.05 as statistically significant.

## Results

The baseline characteristics between the case and control individuals are shown in Table 1. The cases were older, and with lower BMI than the controls. There was a higher proportion of ever smokers among cases compared to controls (31.4% vs 16.7%). And the smoking index of the case group was 1.3 times that of the control group. There were no differences in distribution of gender and alcohol drinking between cases and controls.

**Table 1:** The baseline characteristics of study population

Variable	Controls	Cases	Total	*P*-value
N	257	207	464	
Age, year	52.7 (7.5)	58.8 (7.4)	55.4 (8.1)	< 0.001
Males, n (%)	91 (35.4)	89 (43.0)	180 (38.8)	0.116
BMI, kg/m^2^	24.9 (3.3)	24.1 (3.4)	24.5 (3.3)	0.016
Ever Smoker, n (%)	43 (16.7)	65 (31.4)	108 (23.3)	< 0.001
Smoking Index*, cigarette-years	560.4 (293.7)	743.1 (509.6)	670.4 (444.2)	0.033
Alcohol Drinker, n (%)	55 (21.4)	60 (29.0)	115 (24.8)	0.076

The continuous and categorical variables are presented as mean and percentages, respectively

BMI, body mass index

Smoking index = the number of cigarettes smoked per day × the number of years of smoking

*: just for ever smokers

**Fig. 2:**
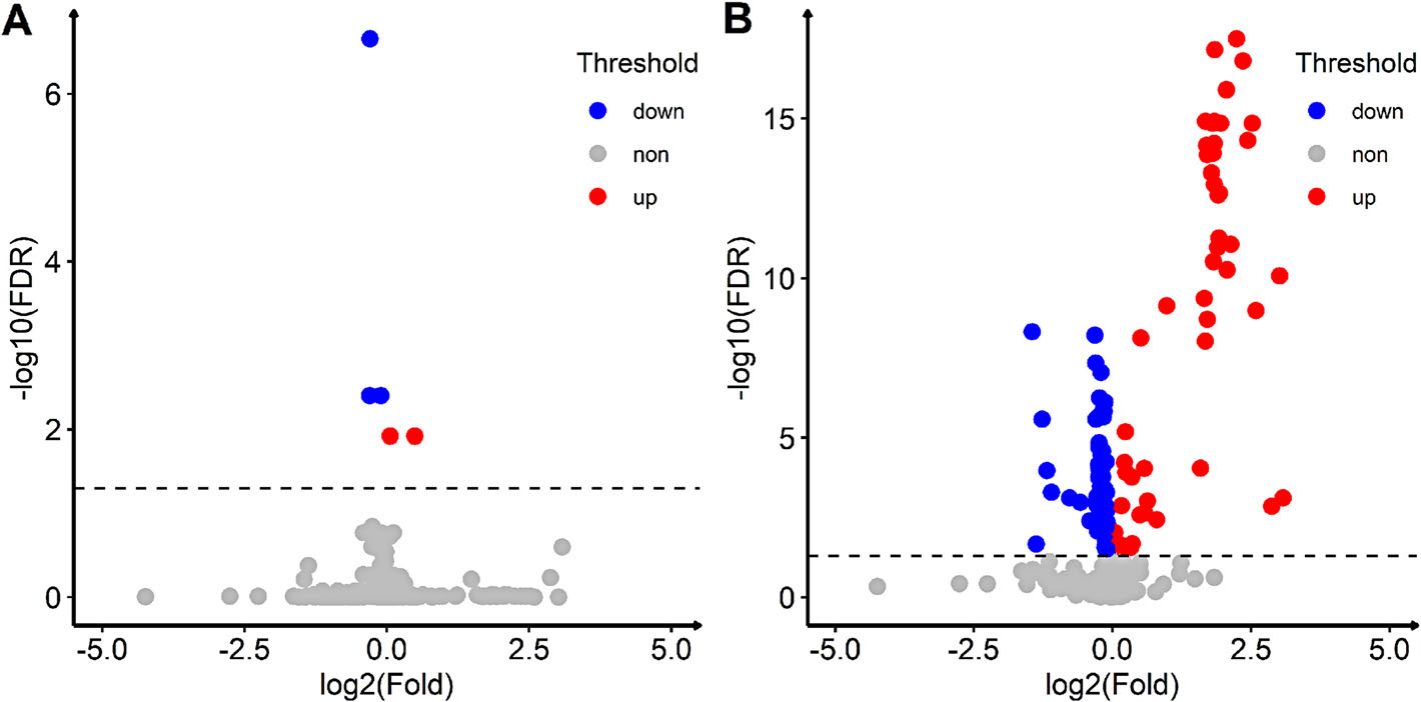
Volcano plot of smoking index and metabolites & ESCC and metabolites (Adjusted for age and gender). **a**: association of smoking index and metabolites; **b**: association of ESCC and metabolites. Fold is the ratio of the mean metabolite content between the case group and the control group. The blue dots indicate FDR <  0.05 and fold < 1, and the red dots indicate FDR <  0.05 and fold > 1. The dashed line is the borderline of FDR = 0.05

Multiple logistic regression suggested that ever smoking was associated with a 3.11-fold increase of ESCC risk (OR = 3.11, 95% CI 1.63–6.05, *P* <  0.001), and for each cigarette-years increase in smoking index, ESCC risk increased by 56% (OR = 1.56, 95% CI 1.18–2.13, *P* = 0.003). There were total 5 metabolites associated with smoking index, among which, compared with the control group, histidine, glutamine and N1-Methyl-2-pyridone-5-carboxamide were down-regulated and PG (10:0/11:0) and PC (14:1/22:2) were up-regulated (Fig. 2a). Associations of metabolites with ESCC are presented in Fig. 2b. Compared with the control group, 53 metabolites were up-regulated and 71 metabolites were down-regulated.

Table 2 shows the selected potential intermediate metabolites of smoking exposure on ESCC by high-dimensional mediation analysis. For ever smoking, a total of 3 metabolites were screened according to FDR less than 0.05, including carnitine (9:0), histidine, and glutamine. The mediation effects were 13.53, 24.80 and 26.58% for carnitine (9:0), histidine and glutamine, respectively. In addition, for cumulative smoking exposure, 5 potential intermediate metabolites were selected as potential mediators. Besides the three metabolites screened by ever smoking, PG (14:1/7:0) and cholic acid were selected as potential mediators as well. The mediation effects of the 5 metabolites were 7.87, 35.51, 29.54, 10.78 and 21.01%, respectively. And the mediation effects of cholic acid, histidine and glutamine were the largest (The overall > 80%). Except PG (14:1/7:0) was positively associated with smoking index and ESCC, the other four metabolites were negatively correlated in both the former and latter paths of the mediation effect.

**Table 2:** The selected potential intermediate metabolites of smoking exposure on ESCC by high-dimensional mediation analyses

Metabolites	α	β	α*β	TE	%TE	FDR
**Ever smoking**						
Carnitine (9:0)	−0.278	−0.552	0.153	1.133	13.53	0.022
Histidine	−0.377	−0.745	0.281	1.133	24.80	0.017
Glutamine	−0.223	−1.349	0.301	1.133	26.58	0.012
**Smoking index**						
PG (14:1/7:0)	2.45E-04	0.549	1.35E-04	1.25E-03	10.78	0.037
Carnitine (9:0)	−2.60E-04	−0.377	9.82E-05	1.25E-03	7.87	0.037
Histidine	−3.45E-04	−1.283	4.43E-04	1.25E-03	35.51	0.008
Glutamine	−2.32E-04	−1.590	3.69E-04	1.25E-03	29.54	0.001
Cholic acid	−4.57E-03	−0.057	2.62E-04	1.25E-03	21.01	0.037

α: represents changes in the former path of the mediation effect, adjusted for age and gender

β: represents changes in the latter path of the mediation effect, adjusted for age and gender

α*β: represents the mediation effect, adjusted for age and gender

TE, total effect

% TE: the proportion of total effect explained by each mediator, calculated as α*β/TE

FDR, false discovery rate

In order to further confirm the above results, we performed univariate mediation analysis on selected metabolites, adjusted for age and gender. Table 3 shows the univariate mediation analyses of smoking exposure on the ESCC risk. For ever smoking, significant positive natural indirect effect (NIE) of histidine and glutamine were observed (*P* <  0.01), and the proportion of mediation were 40.47 and 30.00%, respectively. However, non-significant NIE was observed for carnitine (9:0). With regard to smoking index, mediation effects of histidine and glutamine were similar to ever smoking, and mediation effect of cholic acid was found as well (8.98%, *P* = 0.02). However, no significant mediation effects on risk of ESCC were observed for PG (14:1/7:0) and carnitine (9:0).

**Table 3:** Univariate mediation analysis of selected metabolites, adjusted for age and gender

Metabolites	NDE	*P*_NDE_	NIE	*P*_NIE_	TE	*P*_TE_	%TE
**Ever smoking**							
Carnitine (9:0)	1.011	0.010	0.103	0.141	1.114	0.003	9.25
Histidine	0.896	0.051	0.609	0.002	1.505	< 0.001	40.47
Glutamine	0.847	0.032	0.363	0.001	1.210	0.002	30.00
**Smoking index**							
PG (14:1/7:0)	1.09E-03	0.008	2.18E-04	0.345	1.31E-03	0.003	16.64
Carnitine (9:0)	1.15E-03	0.008	1.12E-04	0.064	1.27E-03	0.004	8.82
Histidine	8.62E-04	0.059	5.74E-04	< 0.001	1.44E-03	0.002	39.86
Glutamine	9.54E-04	0.031	3.97E-04	< 0.001	1.35E-03	0.002	29.41
Cholic acid	1.15E-03	0.008	1.14E-04	0.016	1.27E-03	0.003	8.98

NDE, natural direct effect

NIE, natural indirect effect

TE, total effect

% TE: the proportion of total effect explained by each mediator, calculated as NIE/TE

**Fig. 3:**
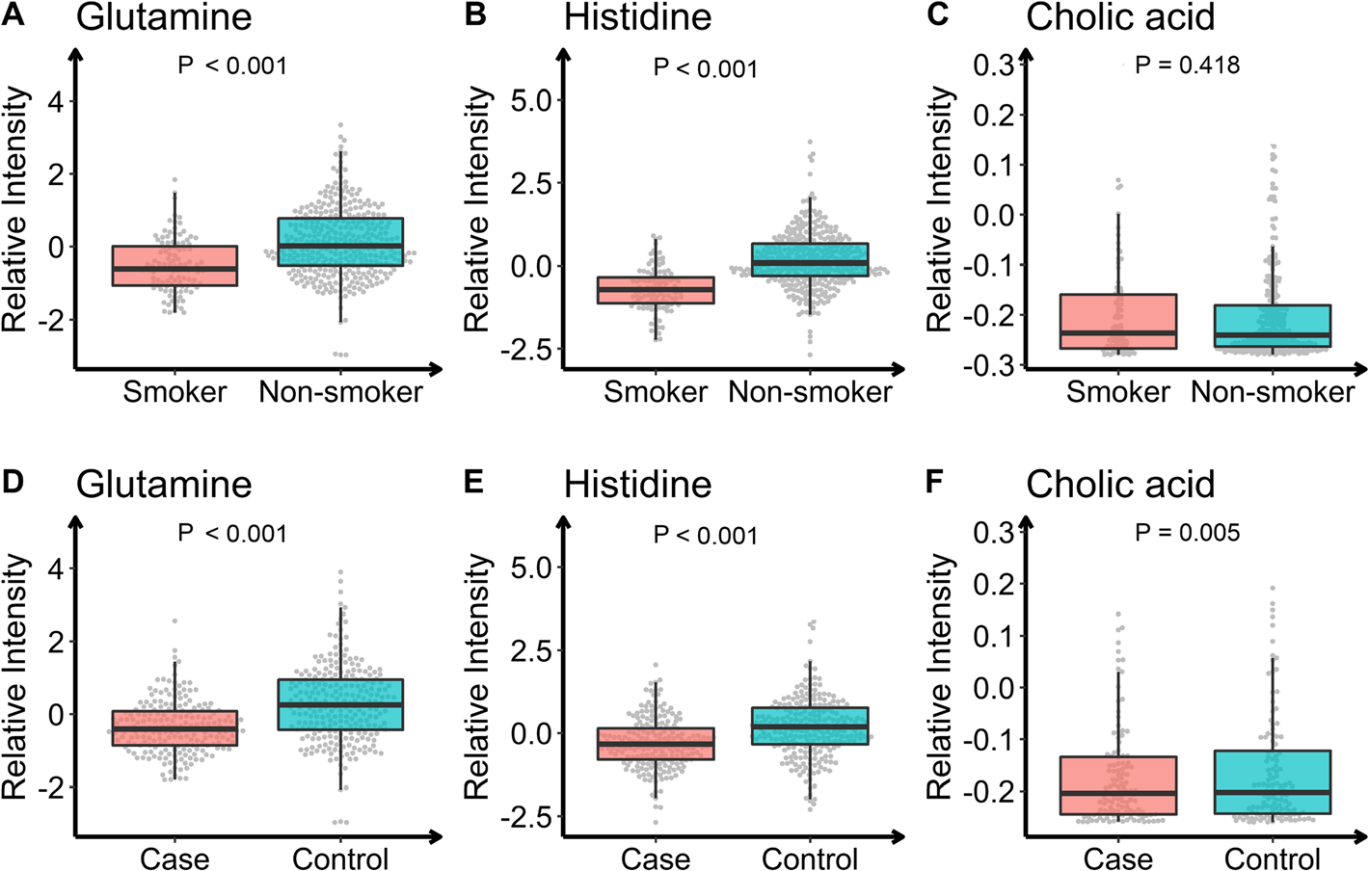
Changing patterns of intermediate metabolites from non-smoker to smoker (**a-c**), and control to case (**d-f**). The relative intensity of each metabolite was standardized with Z-transformation (mean = 0, SD = 1)

We further compared the intergroup differences of the three metabolites in ever smoking and pathological outcomes. Figure 3 shows the differences of metabolites between smokers and non-smokers, and between the case and control groups. The relative intensity of each metabolite was standardized with Z-transformation (mean = 0, SD = 1). Glutamine and histidine were down-regulated in smokers, whereas cholic acid did not show statistical difference between the two groups. And all three metabolites were down-regulated in the case group, consistent with the results of high-dimensional mediation analysis.

## Discussion

In the current study, we assessed the association of smoking exposure with ESCC risk and potential metabolomics mechanisms mediating this association. To our knowledge, this is the first study to investigate the mediation effects of metabolites in the association between cigarette smoking exposure and ESCC risk. Significantly increased risk of ESCC caused by ever smoking and smoking index were observed, though the effects were not exactly the same. For ever smoking, we observed significant natural indirect effects of histidine and glutamine on ESCC risk. We also found the mediation effect of cholic acid in the analysis of smoking index. These results suggest that the smoking index is an important indicator that complements the results of ever smoking.

Smoking, a risk factor for many diseases, is also considered a key risk for ESCC. In this study, for each cigarette-years increase in smoking index, ESCC risk increased by 56%, and the risk of ever smoking for ESCC is higher (OR = 3.11, 95% CI 1.63–6.05), which was consistent with previous studies [27, 28]. Then, we found that the metabolites related to smoking index mainly included histidine and glutamine, and there were more ESCC-related metabolites.

Pre-existing studies have identified a series of differential metabolites between ESCC and normal group, indicating that metabolites may play a critical role in ESCC [8–10]. In this study, we identified 3 and 5 metabolites as the mediators by the high-dimensional mediation analysis of ever smoking and smoking index, respectively. For ever smoking, glutamine and histidine explained 50% of the total effect, while carnitine (9:0) is lower than glutamine and histidine. And when it comes to smoking index, PG (14:1/7:0) and cholic acid were screened as potential intermediate metabolites as well. Our subsequent univariate mediation analysis further proved that histidine, glutamine, and cholic acid may be the intermediate variables. In addition, these 3 metabolites were down-regulated in smokers and case group, except for cholic acid in smokers. And which was consistent with the results of high-dimensional mediation analysis. However, to date, the association of metabolites with ESCC risk has been inconclusive. By showing significant mediation effects of these three metabolites, we consider that our study provides additional evidence for the temporal relationship between metabolites and disease which is in line with previous research hypothesis.

Nicotine is the primary component of cigarette that maintains the smoking habit and develops an addiction. Several substances participate in the addictive effects of nicotine including glutamate, cannabinoids, gamma-aminobutyric acid, and opioids [29]. Glutamine, an amide of glutamate, a non-essential amino acid, can be converted from glucose in vivo [30]. The glutamate pathway mechanism of nicotine addiction can be explained as nicotine induces glutamate release from the prefrontal cortex [31], glutamate as an excitatory neurotransmitter and binds to acetylcholine receptors, causing an increase in dopamine release to produce a reward effect [32, 33]. Repeated exposure to nicotine leads to neural adaptation, thus result in dependence and tolerance [34]. Once without nicotine exposure, withdrawal symptoms will occur [35]. An experimental study found glutamine supplementation could attenuate loss of protein in the muscle in tumor-bearing animals and protect immune and gut-barrier function during radio-chemotherapy in patients with esophageal cancer [36], and another study also found a protective effect of glutamate [37]. In the current study, we demonstrated that lower glutamine is associated with a subsequent occur of ESCC, supports these studies.

The fragile histidine triplet is a tumor suppressor gene, encodes the trivalent histidine (histidine triplet) domain, an abnormality of which will leads to reduced histidine transcription [38, 39]. Previous studies have found abnormal methylation of the fragile histidine triplet gene can lead to ESCC [40], and the abnormal methylation can be induced by cigarette smoking [41]. These findings suggest that cigarette smoking leads the fragile histidine triplet gene abnormal methylation, which reduces histidine transcription, that is, a decrease in histidine in the body, then leads to ESCC. And our study found that histidine was higher in non-smokers than in smokers, supported this conclusion.

Cholic acid is a metabolite of cholesterol, including cholic acid, deoxycholic acid, goosedeoxycholic acid, and ursodeoxycholic acid, which combines with amino compounds to form bile acid [42]. Reflux of bile acid into the esophagus induces esophagitis, inflammation-stimulated hyperplasia, metaplasia such as barrett’s esophagus, and esophageal adenocarcinoma [43, 44]. One study found cigarette smoking aggravates esophageal epithelial changes caused by bile acid reflux [45]. Meanwhile, nicotine could induce cell damage by a multiple stress inducer, deoxycholate [46], and an animal study showed that ursodeoxycholic acid could prevent esophageal adenocarcinoma [47]. Therefore, we speculate that smoking aggravates bile acid reflux, which reduces cholic acid in the blood and further causes ESCC, and appropriate supplement of cholic acid can protect ESCC. And our study found that cholic acid in non-smokers was higher than that in smokers, suggests that this is a possible explanation. And, further research is needed to confirm our findings.

A strength of this study is that it is the first study to investigate the mediation effects of metabolites in the association between cigarette smoking exposure and ESCC risk. In addition, we identified 3 potential mediators, which may provide evidence for the pathogenesis of ESCC and provide new targets for the intervention of ESCC.

Several potential limitations also warrant mention. First, we don’t have information on other potential confounding factors, which may introduce bias. Second, we only analyzed metabolite peaks identified by in-house standard MS/MS spectral library and online databases, which may omit potential intermediate metabolites. However, we believe that it is more significant to use metabolites that are easy to detect and identify for the etiology study and prevention study of disease. Third, since there were no additional blood samples for targeted metabolomics analysis and no validation of metabolic profiles, so further biological studies are needed to validate our results. Fourth, this study is a cross-sectional study and cannot determine a causal relationship, so attention should be paid to the interpretation of the results. While mediation analysis is an effective method for causal inference, and our independent variable is long-term smoking exposure within a temporal relation with the occurrence of disease. Finally, for an observational study, further detailed investigation is necessary.

## Conclusions

In conclusion, cigarette smoking is associated with an increased risk of esophageal squamous cell carcinoma. We found 3 serum metabolites (histidine, glutamine, and cholic acid) may mediate this process. And it seems that as these 3 metabolites were reduced, which would promote the development of esophageal squamous cell carcinoma. The study is beneficial to the etiological study of esophageal squamous cell carcinoma, whereas as an observational study, further biological studies are needed to confirm the mechanism of metabolites in cigarette smoking and esophageal squamous cell carcinoma.

## Supplementary Information

**Additional file 1.**

## Data Availability

The datasets generated and/or analysed during the current study are not publicly available due the datasets used in this study contain personal information but are available from the corresponding author on reasonable request.

## Abbreviations

ESCC: Esophageal squamous cell carcinoma
BMI: Body mass index
QC: Quality control
OR: Odds ratio
CI: Confidence interval
FDR: False discovery rate
NIE: Natural indirect effect
NDE: natural direct effect
TE: total effect
