# Supplementary material for: The mediation effect of serum metabolites on the relationship between long-term smoking exposure and esophageal squamous cell carcinoma
Source: BMC Cancer. 2021 Apr 15;21:415. doi: 10.1186/s12885-021-08151-6 (PMC8050928; doi:10.1186/s12885-021-08151-6)
Supplement: Supplementary file 1 — Additional file 1. [file 12885_2021_8151_MOESM1_ESM.docx]

**Supplement materials**

**The mediation effect of serum metabolites on the relationship between long-term smoking exposure and esophageal squamous cell carcinoma**

Mengke Wei^1^, Lihong Zhao^2^, Jiali Lv^1^, Xia Li^1,3^, Guangshuai Zhou^1^, Bingbing Fan^1^, Xiaotao Shen^4^, Deli Zhao^2^, Fuzhong Xue^1^, Jialin Wang^1,3^, Tao Zhang^1^

1 Department of Biostatistics, School of Public Health, Cheeloo College of Medicine, Shandong University, Jinan, Shandong, 250012, China

2 Tumor Preventative and Therapeutic Base of Shandong Province, Feicheng People’s Hospital, Feicheng 271600, China

3 Shandong Cancer Hospital and Institute, Shandong First Medical University and Shandong Academy of Medical Sciences, Jinan 250117, China

4 Interdisciplinary Research Center on Biology and Chemistry, and Shanghai Institute of Organic Chemistry, Chinese Academy of Sciences, Shanghai 200032, China

**Corresponding author:**

Tao Zhang, MD, PhD

Department of Biostatistics, School of Public Health, Cheeloo College of Medicine, Shandong University, Jinan, Shandong, 250012, China

PO Box 100, 44 Wenhua Xi Rd, Jinan 250012, China.

Fax: 0531-88382140

Email: taozhang@sdu.edu.cn

**Sample preparation**

Serum samples were thawed at 4 ˚C on ice. Then 50 μL of serum sample was taken and placed in a 96-well plate, then extracted with 150 μL of MeOH (which was kept at -20 ˚C before extraction) using Bravo liquid handling system (Agilent Technologies, USA), and followed by vortex for 30 s and incubation for 2 h at -20 ˚C to precipitate proteins. The 96-well plate was then centrifuged at 4000 rpm for 20 min at 4 ˚C. The resulting supernatants were transferred to LC–MS vials and stored at -80 ˚C until the UHPLC-QTOF/MS analysis.

**UHPLC-QTOF/MS analysis**

The serum samples were randomly injected for the UHPLC-QTOF/MS analysis. Quality control (QC) samples were prepared by pooling aliquots of all serum samples that were representative of the serum samples under analysis, and used for data normalization. Blank samples (75 % ACN in water) and QC samples were injected every eight samples during acquisition.

The UHPLC-QTOF/MS analyses were performed using a UHPLC system (1290 series, Agilent Technologies, USA) coupled to a quadruple time-of-flight (QTOF) mass spectrometer (Agilent 6550 iFunnel Q-TOF, Agilent Technologies, USA). Waters ACQUITY UHPLC HSS T3 columns [particle size, 1.8 μm; 100 mm (length) Χ 2.1 mm (i.d.)] were used for the LC separation and the column temperature was kept as 25 ˚C. The flow rate was 0.5 mL/min and the sample injection volume was 6 μL. The mobile phases A was 0.1 % FA in water in positive mode (ESI+) or 0.5 mM NH_4_F in water in negative mode (ESI-), and B was 0.1 % FA in ACN in positive mode or 100 % ACN in negative mode. The linear gradient was set as follows: 0–1 min: 1 % B, 1–8 min: 1 % B to 100 % B, 8–10 min: 100 % B, 10–10.1 min: 100 % B to 1 % B, 10.1–12 min: 1 % B. The acquisition rate was set as 4 spectra/s and the TOF mass range was set as m/z 50–1200 Da. The parameters of MS data acquisition were set as follows: sheath gas temperature, 400 ˚C; dry gas temperature, 250 ˚C; sheath gas flow, 12 L/min; dry gas flow, 16 L/min; capillary voltage, 3000 V in positive mode or -3000 V in negative mode, respectively; nozzle voltage, 0 V; and nebulizer pressure, 20 psi in positive or 40 psi in negative mode, respectively.

Tandem mass spectrometry (MS/MS) data acquisition was performed using another quadruple time-of-flight mass spectrometer (Triple TOF 5600+, AB SCIEX, USA). QC samples were used for MS/MS data acquisition. To expand the coverage of MS/MS spectra, the mass range were divided into four segments: 50–300 Da, 290–600 Da, 590–900 Da, 890–1200 Da. The acquired MS/MS spectra were matched against in-house tandem MS spectral library for metabolite identification. The source parameters were set as follows: GAS1, 60; GAS2: 60; CUR: 30; TEM: 600 ˚C; ISVF: 5500 V and -4500 V in positive and negative modes, respectively.

**Data preprocessing and annotation**

MS raw data (.d) files were converted to the mzXML format using ProteoWizard, and processed by R package XCMS (version 3.2). The preprocessing results generated a data matrix that consisted of the retention time (RT), mass-to-charge ratio (m/z) values, and peak intensity. R package CAMERA was used for peak annotation after XCMS data processing. Metabolic features detected less than 80% in all the QC samples were discarded. Only monoisotopic peaks annotated by CAMERA were selected for the subsequent statistical analyses.

**Metabolite identification**

Tandem mass spectrometry (MS/MS) spectra data for metabolites were acquired using AB Sciex TripleTOF 5600+. The acquired MS/MS spectra were matched against our in-house standard MS/MS spectral library and metabolite standards. The MS/MS spectra match score was calculated using dot-product algorithm ranging from 0-1. The cutoff for match score was set as 0.8. The MS/MS spectra match results were further manually checked to confirm the identification. All the m/z errors are less than 25 ppm and all the RT errors are less than 18 seconds.

There are two lipids that were identified through manual analysis of accurate mass and MS/MS spectra. In brief, characteristic ion of phosphatidylcholine (184.0739, positive mode) was found in both MS/MS spectra, therefore, the two metabolites were identified as phosphatidylcholine (PC). The search of accurate mass against all PC lipids identifies the two lipids as PC (14:0/0:0) and PC (16:0/18:2). Then the chemical structures of other fragments were also inferred to further confirm the structure of PC (14:0/0:0) and PC (16:0/18:2).

Secondly, the biomarkers whose MS/MS spectra were not matched in our in-house databases or cannot be interpreted by MS/MS spectra, were searched against online databases of HMDB ([www.hmdb.ca](http://www.hmdb.ca/)), METLIN (<http://metlin.scripps.edu/>) and KEGG (<http://www.genome.jp/kegg/>). The mass tolerance between the measured m/z values and the exact masses of the components of interest was set to within 30 ppm. The potential biomarkers were identified according to their molecular weights.
